# Effects of Dietary Fish Oil Supplementation on the Growth, Proximate Composition, and Liver Health of Chinese Stripe-Necked Turtle (*Mauremys sinensis*)

**DOI:** 10.3390/ani14172511

**Published:** 2024-08-29

**Authors:** Juntao Li, Yaopeng Lu, Huiqin Chen, Peihua Zheng, Xiuxia Zhang, Zelong Zhang, Li Ding, Dongmei Wang, Chi Xu, Xiaoqi Ai, Qiongyu Zhang, Jianan Xian, Meiling Hong

**Affiliations:** 1 Ministry of Education Key Laboratory for Ecology of Tropical Islands, Key Laboratory of Tropical Animal and Plant Ecology of Hainan Province, College of Life Sciences, Hainan Normal University, Haikou 571158, China; lijuntao@itbb.org.cn (J.L.); dingli@hainnu.edu.cn (L.D.); 15501966265@163.com (X.A.); 18189887514@163.com (Q.Z.); 2 Hainan Provincial Key Laboratory for Functional Components Research and Utilization of Marine Bio-Resources, Institute of Tropical Biosciences and Biotechnology, Chinese Academy of Tropical Agricultural Sciences, Haikou 571101, China; luyaopeng@itbb.org.cn (Y.L.); chenhuiqin@itbb.org.cn (H.C.); zhengpeihua@itbb.org.cn (P.Z.); zhangxiuxia@itbb.org.cn (X.Z.); zhangzelong@itbb.org.cn (Z.Z.); wangdongmei@itbb.org.cn (D.W.); xc18551892763@163.com (C.X.)

**Keywords:** *Mauremys sinensis*, fish oil level, proximate composition, immunity, apoptosis

## Abstract

**Simple Summary:**

In this study, the effects of fish oil supplementation on the growth performance, physiological and biochemical indicators, immune function, and liver cell health of the Chinese stripe-necked turtle (*Mauremys sinensis*) were examined. Increasing the level of fish oil in the diet improved the growth performance of *M. sinensis* and increased the lipid content in the body, but it induced oxidative stress in the liver. Furthermore, it decreased the immune function of *M. sinensis*, increased instances of apoptosis in liver cells, and caused liver damage. These results highlight the importance of identifying the appropriate lipid supplementation level to promote growth and maintain health.

**Abstract:**

Dietary lipids provide energy for animals and can also be converted into other nutrients (such as non-essential amino acids), which play a role in saving protein. The Chinese stripe-necked turtle is a protected and endangered species that has been bred in captivity; however, basic data on lipid requirements remain unavailable. In this study, 360 *Mauremys sinensis* (body weight of 65.32 ± 0.15 g) were randomly divided into six groups with three replicates per group; the turtles were fed experimental diets supplemented with various levels of fish oil (i.e., 1% (control group, CG), 3.5% (HF-1), 6% (HF-2), 8.5% (HF-3), 11% (HF-4), and 13.5% (HF-5)) for 10 weeks. The results showed that compared with CG, increasing the fish oil level promoted the growth performance of turtles, and the HF-3 group achieved the best effect. The HF-4 group showed the highest increases in the hepatosomatic index and viscerosomatic index. In addition, increased lipid levels also increased the crude lipid content and reduced the crude protein content in muscle tissue. Oil red O staining showed that the liver lipid content increased with the level of supplemented fish oil, which is consistent with the results of the hepatosomatic index. Compared with CG, triglyceride, total cholesterol, and low-density lipoprotein cholesterol increased significantly in both the liver and serum when fish oil levels exceeded 8.5% (*p* < 0.05), while high-density lipoprotein cholesterol decreased significantly. Aspartate transaminase and cerealthirdtransaminase levels in serum increased significantly when fish oil levels exceeded 8.5% (*p* < 0.05). Moreover, the activities of antioxidant enzymes (GSH-Px, SOD, T-AOC, and CAT) and MDA showed similar results, indicating that high fish oil levels (8.5–13.5%) caused liver tissue damage in *M. sinensis*. Increased fish oil levels significantly upregulated the expression levels of cytokines (*IFN-γ*, *TNF-α*, *TGF-β1*, *IL-10*, and *IL-12*) (*p* < 0.05), downregulated the expression levels of antioxidant enzyme-related genes (*cat*, *mn-sod*, and *gsh-px*), and increased apoptosis of liver cells. Supplementation of the diet with 3.5–6% fish oil improved the growth performance of *M. sinensis*, and the turtles maintained a beneficial immune status. The results provide a scientific basis for optimizing the commercial feed formula of *M. sinensis*.

## 1. Introduction

The Chinese striped-necked turtle (*Mauremys sinensis*) is a rare freshwater turtle species, distributed in the southern reaches of the Yangtze River in China, including the province of Hainan as well as Taiwan. It is one of the most widely distributed and abundant species of freshwater turtles in China [1,2]. Because of its high economic value for medicinal, culinary, and ornamental purposes [3], the wild population of *M. sinensis* is currently critically endangered and continues to decline fueled by overhunting [4,5]. Under natural living conditions in the wild, *M. sinensis* is omnivorous, and both plants and small animals are its common food sources. Artificial breeding is mainly based on commercial feed. Previous studies on *M. sinensis* mainly focused on aquaculture and breeding [6], environmental toxicology [7,8], and physiological ecology [9,10]. However, few studies examined the dietary nutrition of *M. sinensis*. For example, whole fish oil, 66.7% fish oil, 33.3% fish oil, and whole soybean oil were used, and the intestinal morphology and microflora composition were examined to assess the lipid requirements of *M. sinensis* [11]. A further study found that with an increasing replacement ratio of soybean oil, the total cholesterol (TC) level exceeded that of the whole fish oil group; furthermore, the total lipase activity of the 66.7% soybean oil replacement group exceeded that of the whole fish oil group. Among these groups, a fish oil–soybean oil ratio of 1:2 was found to yield the best replacement effect [12]. In addition, feeding different proportions of lipid sources changed the composition of fatty acids in different tissues of *M. sinensis* [13]. However, systematic studies on lipid requirements are still rare.

Lipids in feed provide energy, phospholipids, and lipid-soluble components and can be converted into other nutrients [14]. Compared with high-quality proteins, lipids are usually available at a relatively low price. A proper lipid content can improve the feed efficiency of farm animals and reduce the need for protein such as fish meal [15]. However, the huge increase in aquaculture has resulted in a shortage of fish meal, which has increased its price considerably. To reduce costs and the use of protein, feed companies prefer to use high-lipid aquatic feed. However, increased lipid levels in the diet of aquatic species can induce a disturbance in lipid digestion and absorption; moreover, when lipids such as triglycerides accumulate in the body, they can induce diseases (e.g., obesity and non-alcoholic lipid liver disease) [16], thereby threatening the health of aquatic species [17]. Previous studies have found that high-lipid feed (13.89% lipid) can cause lipid accumulation in the liver of the Chinese softshell turtle (*Pelodiscus sinensis*) [18] and increase the lipid content in the muscle tissue of the grass carp (*Ctenopharyngodon idella*) [19]. Moreover, 18.08% lipid in the diet increased the apoptosis of liver cells of largemouth bass (*Micropterus salmoides*) [20]; 10.1% dietary lipid caused oxidative stress in the blunt snout bream (*Megalobrama amblycephala*) [21]; 17% dietary lipid increased the inflammatory response of the blackhead seabream (*Acanthopagrus schlegelii*) [22]; and 12.5% dietary lipid downregulated the fat synthesis gene and changed the bile acid composition in vivo in the Japanese puffer (*Takifugu rubripes*) [23]. The specially formulated feed for farmed turtles has problems associated with excessive lipid addition. Excessive lipid deposition in the liver can cause liver damage including inflammatory responses and apoptosis. To evaluate the degree of liver damage, the expression levels of key genes involved in inflammation (*IFN-γ*, *TNF-α*, *TGF-β1*, *IL-6*, *IL-10*, and *IL-12*) and apoptosis (*Caspase-3*, *BAX*, and *Bcl-2*) are usually assessed. Based on the above, it is meaningful to study the lipid requirement of *M. sinensis* to obtain a more balanced feed formula and to enhance the health status of *M. sinensis*, which will benefit artificial protection efforts.

## 2. Materials and Methods

### 2.1. Diet Preparation

In the feed formula, the protein sources were fish meal and soybean meal, the sugar source was flour, and the main source of lipids was fish oil. Six diets were configured according to different proportions of added fish oil while controlling the protein level of each diet at 37%. The supplemented levels of fish oil in the six groups were 1% (CG), 3.5% (HF-1), 6% (HF-2), 8.5% (HF-3), 11% (HF-4), and 13.5% (HF-5). The supplemented level of 1% fish oil in CG was set based on a prior study [11]. All ingredients were passed through an 80-mesh sieve, mixed thoroughly, and extruded into 2.5 mm diameter pellet feed by a DGP40-B feed machine (Yutian Machinery Co., Ltd., Xingtai, China). Finally, the pellets were dried in a ventilated place and stored in a freezer at −40 °C until use. The composition and approximate composition of the feed formula are shown in Table 1.

### 2.2. Experimental Animals and Feeding Management

The one-year-old *M. sinensis* individuals used in the experiment were purchased from Haikou Hongwang Agricultural Breeding Co., Ltd., Haikou, China. After being domesticated in a breeding room for two weeks, 360 healthy turtles (65.32 ± 0.15 g) were selected as experimental turtles and disinfected with 2% sodium chloride. During the temporary feeding period, all turtles were fed with the same diet as CG for 2 weeks. After that time, the feed was changed to the experimental diets with six different lipid levels. The six groups of *M. sinensis* were raised in different feeding tanks but in the same feeding room. The water in each tank was changed and disinfected regularly using the same disinfectant and water source for all tanks. Each group was equipped with three breeding repeat tanks of 100 × 120 × 80 cm, and 20 *M. sinensis* individuals were placed in each tank. The water was changed every two days, and the amount of water changed each time was about 1/3 of the total amount of water. The culture condition was controlled as follows: the water temperature was 23–25 °C, the pH was 7.2–7.4, the ammonia nitrogen concentration was below 0.02 mg/L, the nitrite concentration was below 0.1 mg/L, and the dissolved oxygen content exceeded 6 mg/L. The feeding amount was 3–5% of the body weight of *M. sinensis*. Feeding was carried out at 6:00 and 18:00 every day, and the amount of feed was adjusted according to the actual feeding requirements. Residual feed was removed 30 min after feeding. The culture experiment lasted for 10 weeks, and samples were taken after the experiment.

### 2.3. Sample Collection and Preparation

After the feeding experiment, *M. sinensis* individuals were fasted for 24 h and dissected under low-temperature anesthesia. The total weight of each group of turtles was measured and recorded before dissection. Six turtles were randomly selected from each tank, and the wet weight of each turtle was measured. Blood was collected, left to stand at room temperature for 2 h, and then centrifuged at 3000 rpm at 4 °C for 15 min. Serum was collected for antioxidant analysis [3]. The liver, leg muscle, and other tissues were removed in turn, and the weights of the liver and other internal organs were recorded. All samples were quickly frozen in liquid nitrogen and transferred to −80 °C for storage. After weighing the total weight of the liver samples, parts were fixed with 4% paraformaldehyde for hematoxylin and eosin staining. The collected samples were quickly frozen in liquid nitrogen and then transferred to −80 °C until further analyses of enzyme activity, physiological and biochemical indexes, body composition, and gene expression.

### 2.4. Growth Target

The weight gain rate (WGR), special growth rate (SGR), survival rate (SR), feed conversion rate (FCR), hepatosomatic index (HSI), and viscerosomatic index (VSI) were calculated as follows [24]:

WGR (%) = 100 × (Final average weight − Initial average weight)/Initial average weight.

SGR (%) = (Ln final average weight − Ln initial average weight) × 100/Breeding days.

SR (%) = 100 × Final mantissa/Initial mantissa.

FCR (%) = Total food intake/(Final average weight − Initial average weight).

HSI (%) = 100 × Hepatopancreas weight/Individual weight.

VSI (%) = 100 × Viscerosomatic weight/Individual weight.

### 2.5. Proximate Composition Analysis

The contents of moisture, crude protein, crude lipid, and ash in the muscle and feed of *M. sinensis* were determined following the method established by the American Association of Official Analytical Chemists [25].

### 2.6. Histological Analysis

After the culture experiment, the liver tissue of each group was dissected, and the lipid content was analyzed. A 2 mm liver tissue sample was cut and washed with 0.1 M phosphate buffer and then snap-frozen in liquid nitrogen. Slices with a thickness of 9 μm were cut and soaked in 1% oil red O working solution for 10 min, hematoxylin was re-dyed, and the samples were rinsed with water for 30 min. The integral optical density of the section field was analyzed under a white light microscope at 200× field of view [20].

### 2.7. Biochemical Determination

Serum biochemical indexes were determined by an automatic physiological and biochemical analyzer (BS-350S, Mindray, Shenzhen, China). The test indexes included the following liver function indexes: cerealthirdtransaminase (ALT), aspartate transaminase (AST), total protein (TP), albumin (ALB), globulin (GLO), total bilirubin (TBIL), direct bilirubin (DBIL), indirect bilirubin (I-BIL), and r-glutamyltransferase (r-GT); the lipid indexes included the following: TC, triglyceride (TG), high-density lipoprotein cholesterol (HDL), low-density lipoprotein cholesterol (LDL), and fasting blood glucose (GLU).

The following biochemical indexes of the liver were determined: ALT (C009-2-1), AST (C010-2-1), TC (A111-1-1), TG (A110-1-1), HDL (A112-1-1), and LDL (A113-1-1). The following immune indexes in the liver and serum were determined: total antioxidant capacity (T-AOC) (A015-2-1) and trace malondialdehyde (MDA) content (A003-2-2), as well as activities of superoxide dismutase (SOD) (A001-3-2), catalase (CAT) (A007-1-1), and glutathione peroxidase (GSH-Px) (A005-1-2). Respective kits were used for these assessments, following the manufacturer’s instructions (Nanjing Jiancheng Bioengineering Institute, Nanjing, Jiangsu, China).

### 2.8. Gene Expression Measurement

Three *M. sinensis* individuals from each experimental group were dissected, and their livers were removed. RNA was extracted with a FastPure Cell/Tissue total RNA isolation kit V2 (Nanjing Vazyme Biotech Co., Ltd., Nanjing, China), and the integrity of total RNA was assessed by 1.2% agarose gel electrophoresis. The concentration and purity of RNA were assessed by a spectrophotometer (NanoDrop Technologies, Wilmington, DE, USA) [3]. Total RNA was reverse-transcribed into cDNA using the PrimeScriptTM RT reagent kit with a gDNA eraser kit (TaKaRa, Code No. RR047A; Takara, Dalian, China). The cDNA template was stored at −80 °C for further experiments.

The β-actin gene was used as a reference gene because of its stable amplification efficiency and dissociation curve. The sequences of relevant primers can be found in previous publications by this research group [2,3,7] and are shown in Table 2. The software Premier v5 (Premier Biosoft International, Palo Alto, CA, USA) was used to design the primers. qRT-PCR was performed on a Stratagene Mx3005P real-time PCR machine (Agilent, Santa Clara, CA, USA) using SYBR Green qPCR Mix (Takara, Dalian, China) as recommended by the manufacturer. The data were processed using the 2^−△△Ct^ method to obtain relative messenger RNA expression levels [26].

### 2.9. Statistical Analysis

The experimental results are presented as mean ± standard error. One-way analysis of variance and Duncan’s multiple comparisons were performed using SPSS version 18.0 (Chicago, IL, USA). *p* < 0.05 indicated a statistically significant difference.

## 3. Results

### 3.1. Growth Performance

Significantly higher WGR and SGR and lower FCR were found in the HF-3 group than in all other groups. Interestingly, HF-4 and HF-5 showed reduced levels of WGR and SGR compared with HF-3. Of these, the WGR and SGR of the HF-3 group were the highest, reaching 95.22 ± 0.73% and 0.96 ± 0.005%, respectively. The FCR of HF-3 was the lowest (1.45 ± 0.009). The HSI levels of HF-3, HF-4, and HF-5 were significantly higher than that of CG, while the VSI levels of the experimental groups (except for HF-1) increased significantly (*p* < 0.05), and in HF-4, both reached the maximum. However, no significant relationship was observed between the level of fish oil and the survival rate (*p* > 0.05; Table 3).

### 3.2. Muscle Composition

The crude lipid content in muscle tissue followed a gradually increasing trend. Compared with CG, the crude lipid content in HF-2, HF-3, HF-4, and HF-5 was significantly higher (*p* < 0.05). The crude protein content showed the opposite trend. At fish oil levels of 11% and 13.5%, the crude protein contents were 79.09 ± 0.44% and 79.31 ± 0.35%, respectively, which were significantly lower than those of CG and other low fish oil groups. However, no significant differences were observed in the ash or moisture contents between CG and the high fish oil groups (*p* > 0.05; Table 4).

### 3.3. Physiological and Biochemical Index Analysis

TG, TC, and LDL showed an increasing trend with increasing levels of fish oil. The levels of HF-3, HF-4, and HF-5 were significantly higher than those of CG (*p* < 0.05), while HDL showed the opposite trend. With changing fish oil level, GLU presented a parabola form, and the highest GLU in HF-3 reached 4.33 ± 0.03 mmol/L, which differed significantly from that in CG (*p* < 0.05). ALT and AST followed a gradual upward trend with increasing fish oil levels, which were significantly higher than those of CG and low-level addition groups at fish oil levels exceeding 11%. The contents of TP, ALB, and GLO in serum showed a trend of first increasing and then decreasing, and the maximum value was observed in HF-3. The TBIL in serum included DBIL and I-BIL. However, no significant difference was found in TBIL or I-BIL among all groups (*p* > 0.05). DBIL in HF-3, HF-4, and HF-5 increased significantly, as in r-GT, with specific differences from CG (Table 5).

With increasing fish oil levels, the levels of TG and TC in the liver increased significantly. A significant difference was observed between CG and the high-level fish oil groups when the fish oil addition exceeded 6% (*p* < 0.05). HDL was higher in the low-level fish oil groups (less than 3.5%) than in the high-level groups. When the levels of supplemented fish oil exceeded 11%, LDL in the liver was significantly lower than in CG (*p* < 0.05). However, no significant difference was found between ALT and AST among the groups with different fish oil levels (*p* > 0.05; Table 6).

### 3.4. Liver Histology

**Figure 1:**
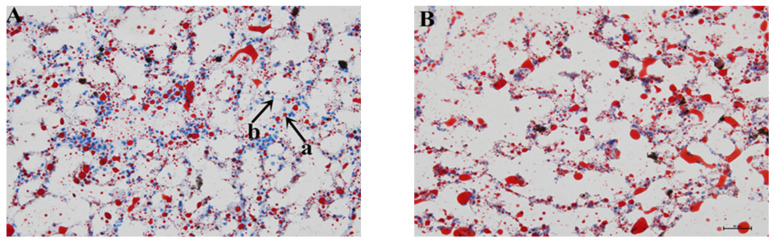

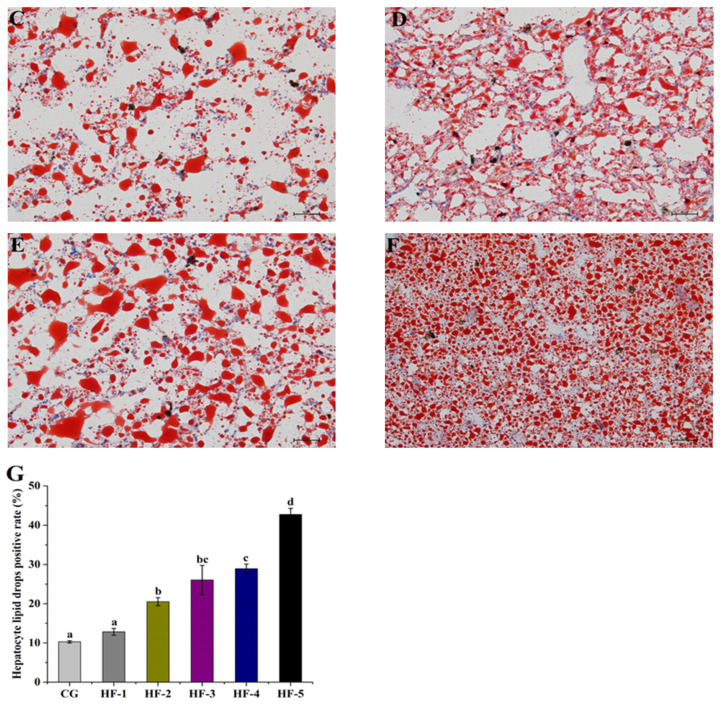
Effect of the fish oil level on lipid accumulation in the liver of *M. sinensis*. Hepatic lipid droplets were visualized by oil red O staining (400× magnification). The red spots are lipid droplets (a); the blue spots are nuclei (b). (**A**) CG (1%); (**B**) HF-1 (3.5%); (**C**) HF-2 (6%); (**D**) HF-3 (8.5%); (**E**) HF-4 (11%); and (**F**) HF-5 (13.5%). (**G**) Lipid accumulation was quantified by measuring the intensity of the stained oil droplets. Values were presented as means ± SEM (*n* = 3) and mean values with unlike letters indicate significant differences (*p* < 0.05).

The nucleus appeared blue in the oil red O staining, while the lipid droplets in cells were stained red. A comparison of the six images shows that the positive rate share of red spots (lipid droplets) increased with increasing supplementation of fish oil. When the fish oil level exceeded 6%, the positive share increased significantly compared with CG (*p* < 0.05). The positive rates of HF-2, HF-3, HF-4, and HF-5 were 20.53 ± 1.02%, 28.92 ± 1.20%, 26.04 ± 3.72%, and 42.76 ± 1.54%, respectively, while that of CG was 10.28 ± 0.29. The blue spots (nuclei) in the same magnification field were scattered and sparse in the high-level fish oil groups, indicating swelling and deformation of liver cells (Figure 1).

### 3.5. Antioxidant Enzyme Activity

**Figure 2:**
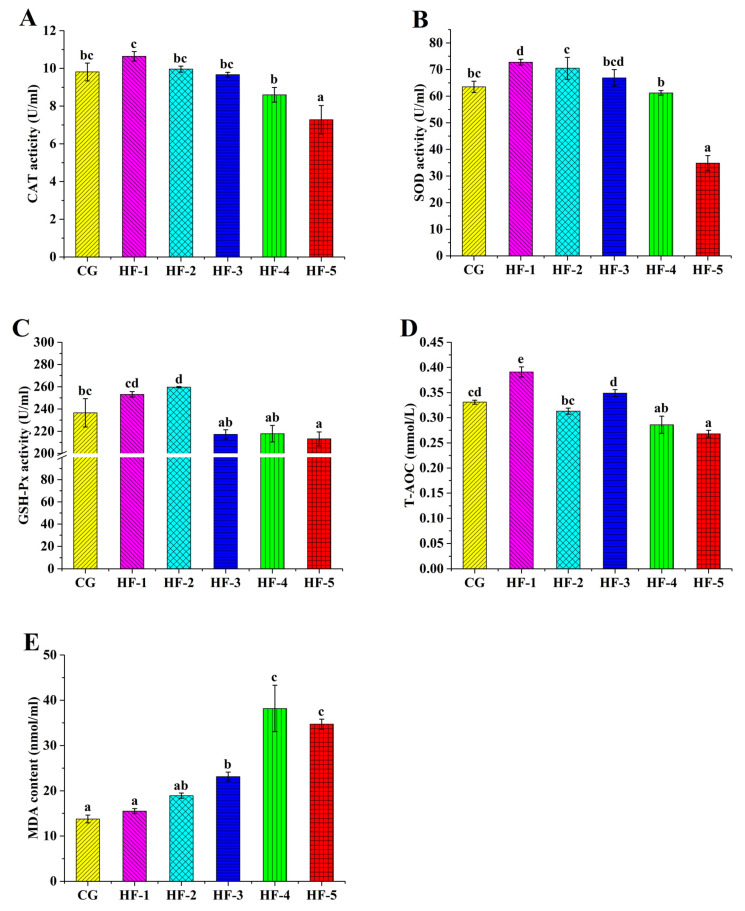
The effect of fish oil levels on antioxidant enzyme activity in the serum of *M. sinensis*. Different letters above the error lines indicate significant differences (*p* < 0.05). (**A**) CAT activity; (**B**) SOD activity; (**C**) GSH-Px activity; (**D**) T-AOC activity; and (**E**) MDA content. CG (1%); HF-1 (3.5%); HF-2 (6%); HF-3 (8.5%); HF-4 (11%); and HF-5 (13.5%) (*n* = 9).

Fish oil supplementation significantly increased the content of MDA in the serum of *M. sinensis*, and significant differences were observed among HF-3, HF-4, HF-5, and CG (*p* < 0.05). The activities of SOD, GSH-Px, CAT, and T-AOC were decreased in the high fish oil groups. The activities of SOD, GSH-Px, and CAT were significantly lower in HF-5 than in CG, while the T-AOC levels in HF-4 and HF-5 were significantly lower than that in CG (*p* < 0.05). The levels of SOD and T-AOC in HF-1 and the level of GSH-Px in HF-2 were significantly higher than those in CG (*p* < 0.05); however, no significant difference was found among other groups (Figure 2).

**Figure 3:**
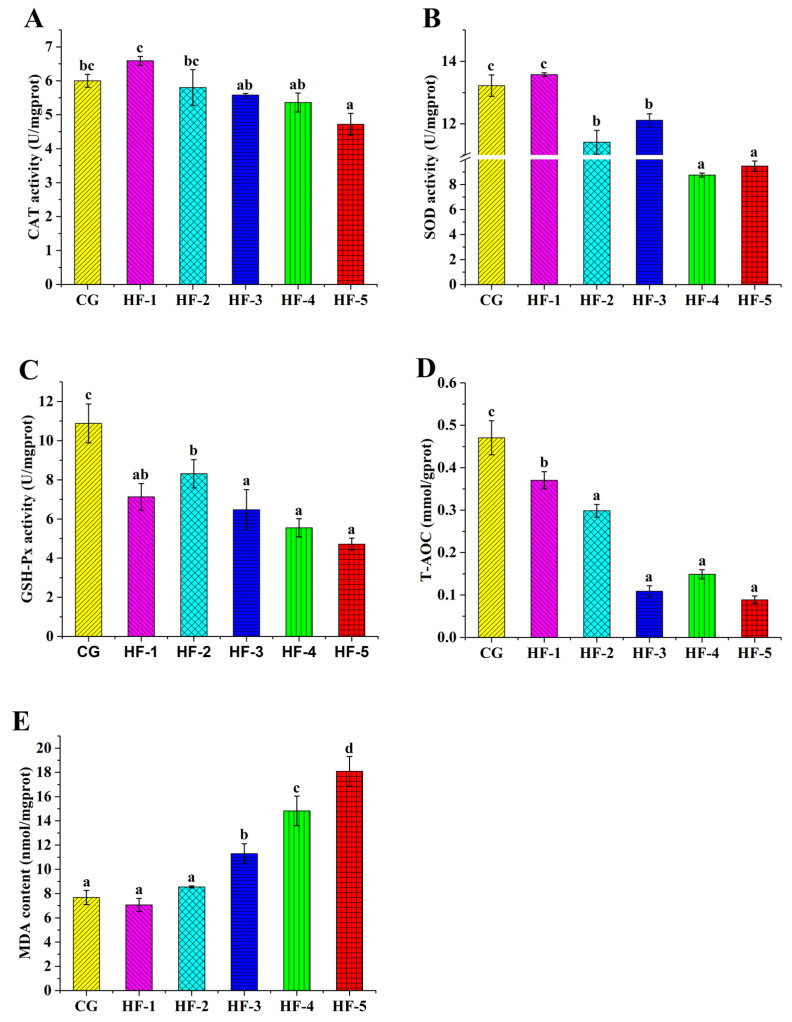
The effect of fish oil levels on antioxidant enzyme activity in the liver of *M. sinensis*. Different letters above the error lines indicate significant differences (*p* < 0.05). (**A**) CAT activity; (**B**) SOD activity; (**C**) GSH-Px activity; (**D**) T-AOC activity; and (**E**) MDA content. CG (1%); HF-1 (3.5%); HF-2 (6%); HF-3 (8.5%); HF-4 (11%); and HF-5 (13.5%) (*n* = 9).

The supplementation of diets with fish oil significantly decreased the activities of SOD, GSH-Px, and CAT in the liver (*p* < 0.05). The T-AOC and GSH-Px levels of all experimental groups were significantly lower than those of CG (*p* < 0.05). The SOD activities in the liver samples of HF-2, HF-3, HF-4, and HF-5 differed significantly from that in CG, while the activity of CAT in HF-5 was significantly lower than that in CG (*p* < 0.05). The MDA contents in the liver samples of HF-3, HF-4, and HF-5 were significantly higher than those of CG, HF-1, and HF-2 (Figure 3).

### 3.6. Expression Levels of Inflammation, Antioxidants, and Apoptosis-Related Genes

#### 3.6.1. Expression of Inflammation Genes

**Figure 4:**
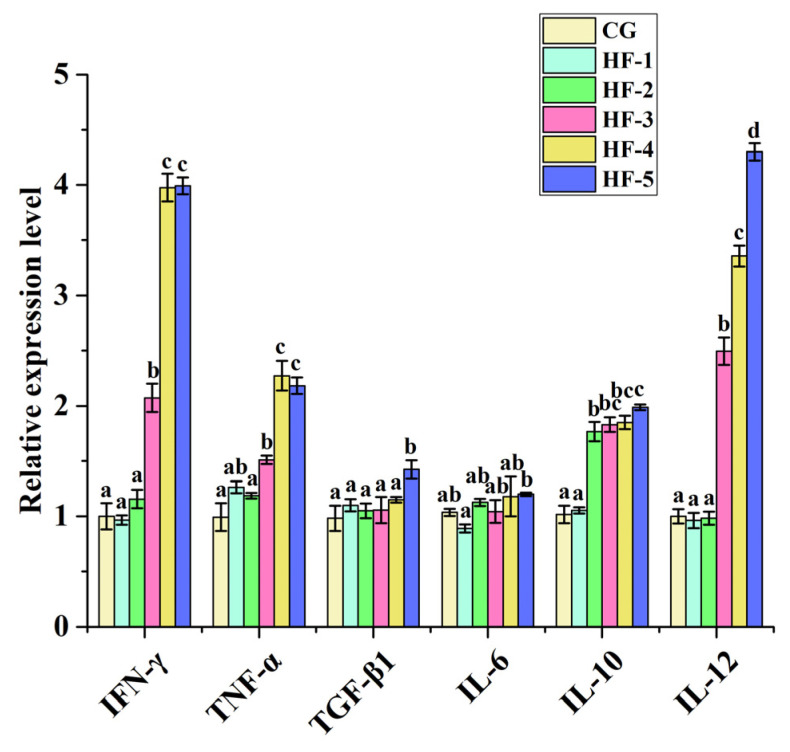
Effect of fish oil levels on the expression level of immunomodulatory genes in the liver of *M. sinensis*. Different letters above the error lines indicate significant differences (*p* < 0.05). CG (1%); HF-1 (3.5%); HF-2 (6%); HF-3 (8.5%); HF-4 (11%); and HF-5 (13.5%) (*n* = 9).

Compared with CG, *IFN-γ*, *TNF-α*, *TGF-β1*, *IL-10*, and *IL-12* were significantly upregulated (*p* < 0.05). The expression levels of *IFN-γ*, *TNF-α*, and *IL-12* were significantly higher in HF-3, HF-4, and HF-5 than in CG, while the expression level of *TGF-β1* was significantly higher in HF-5. The expression level of *IL-10* was significantly higher in the experimental groups than in CG, except for HF-1 (*p* < 0.05) (Figure 4).

#### 3.6.2. Expression of Antioxidant-Related Genes

**Figure 5:**
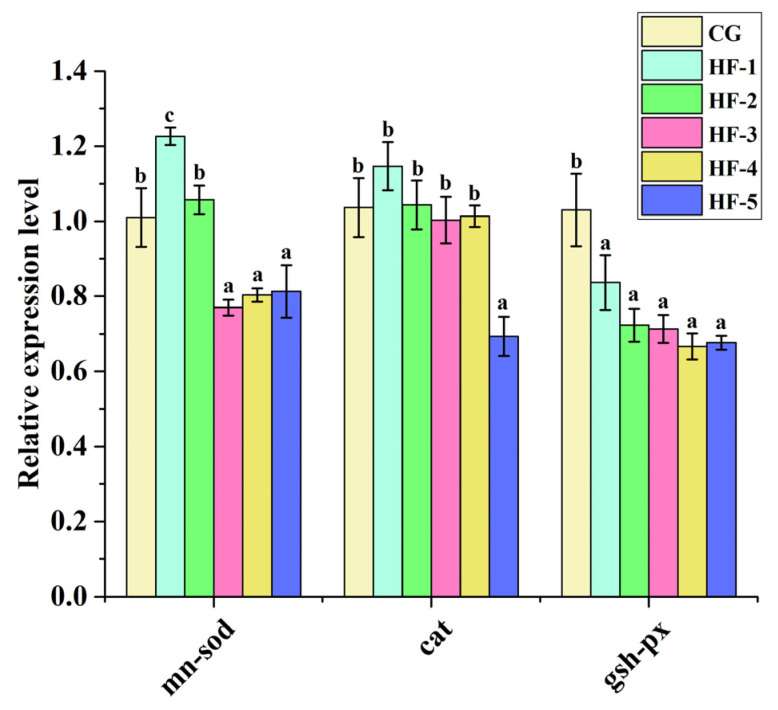
Effect of fish oil levels on the expression level of antioxidant-related genes in the liver of *M. sinensis*. Different letters above the error lines indicate significant differences (*p* < 0.05). CG (1%); HF-1 (3.5%); HF-2 (6%); HF-3 (8.5%); HF-4 (11%); HF-5 (13.5%) (*n* = 9).

Compared with CG, the expression level of *cat* in the liver of HF-5 and the expression levels of *sod* in HF-3, HF-4, and HF-5 were significantly lower (*p* < 0.05). The expression levels of *gsh-px* in all experimental groups were lower than in CG. No significant difference was found among all lipid-supplemented groups (*p* > 0.05; Figure 5).

#### 3.6.3. Expression of Apoptosis-Related Genes

**Figure 6:**
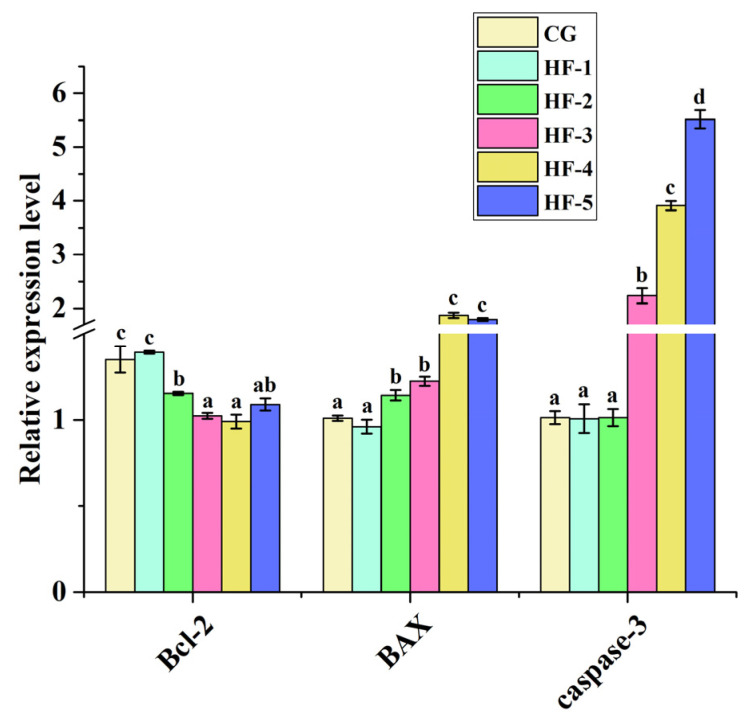
Effect of fish oil levels on the expression level of apoptosis-related genes in the liver of *M. sinensis*. Different letters above the error lines indicate significant differences (*p* < 0.05). CG (1%); HF-1 (3.5%); HF-2 (6%); HF-3 (8.5%); HF-4 (11%); HF-5 (13.5%) (*n* = 9).

Compared with CG, the expression level of *Caspase-3* was significantly upregulated in the liver samples of HF-3, HF-4, and HF-5. Except for HF-1, the expression level of *BAX* was significantly upregulated, and *Bcl-2* was significantly downregulated in other experimental groups compared with CG (*p* < 0.05; Figure 6).

## 4. Discussion

Lipids are required to maintain the normal operation of the body, and the relevant lipid acids are mainly derived via dietary intake. In this study, measured dietary lipid levels ranged from 6.74% to 18.07%. Compared with CG, the increased proportion of fish oil increased WGR and SGR and reduced FC. Similar findings have been reported in previous studies; for example, in zebrafish (*Danio rerio*) fed with high-lipid feed (10% lipids), WGR increased, and obesity was induced [27]. The growth rate and feed efficiency of *Epinephelus fuscoguttatus*♀ × *E. polyphekadion*♂ was improved by increasing the lipid content in diets [28]. In addition, in a study on the European seabass (*Dicentrarchus labrax*), the daily growth rate was positively correlated with the dietary lipid level [29]. Interestingly, in the present study, it was found that both the HSI and VSI of *M. sinensis* in the groups with fish oil levels exceeding 8.5% were significantly higher than those in CG. A study on *M. salmoides* showed that high-lipid feed can improve VSI and abdominal lipid deposition [20]. A similar study was conducted on rainbow trout (*Oncorhynchus mykiss*) fed with a high-lipid diet that resulted in fatty liver as well as increased weight and HSI levels [30]. In a study where large yellow croakers (*Larimichthys crocea*) were fed a high-lipid diet formulated with 6.5% fish oil + 6.5% soybean oil, FC and HSI were significantly elevated [31]. Based on the above, lipids may prefer to deposit in the liver and mesentery, inducing an increase in the related indexes. However, different species may show various levels of lipid tolerance related to various factors (e.g., feed formula, farming cycle, and environmental factors).

In this study, analysis of the muscle composition of *M. sinensis* showed that except for HF-1, the crude lipid content was significantly higher in the muscles of all experimental groups than in CG (*p* < 0.05). The crude protein content in muscle tissue was significantly lower under lipid supplementation, exceeding 11%. Zhou et al. (2017) found that cultivating grass carp with high-lipid feed resulted in higher crude lipid levels in the whole fish, muscle, and liver compared with the levels when fed with a low-lipid diet; however, the muscle tissue had a lower crude protein content [19]. Similar results were obtained in a study on large yellow croaker [31] and European seabass [29]. Interestingly, researchers hold different views on the location of lipid deposits after feeding high-lipid diets. For example, a high-lipid diet increased crude lipids in the whole fish and liver of *E. fuscoguttatus*♀ *× E. polyphekadion*♂ [28]. A high-lipid diet (lipid level 14%) led to an increase in the crude lipid content in the whole fish of *E. fuscoguttatus × E. lanceolatus,* while crude lipid contents did not change significantly in the liver or muscle [15,32]. In a different study, grass carp received diets with low, medium, and high lipid levels; the lipid content in whole fish was positively correlated with dietary lipid levels, but there was no significant difference in liver lipid content [33]. Combined with the results of this study, the possible reasons for these observations include the following: On the one hand, after hydrolysis by pancreatic lipase, feed-derived lipids are emulsified by bile acids in the intestinal lumen, producing sn-2-monoacylglycerol and free fatty acid products [34]. Because nutritionally sound feed formulations were provided, these excess fatty acids could be used to synthesize other complex lipids, which may be stored anywhere in the body. On the other hand, special attention was paid to the living habits of turtles. *M. sinensis* hibernates and uses various parts of its body to store energy during the active growth period; this difference from other aquatic animals also explains the obtained research results.

Usually, the physiological and biochemical indicators of serum and liver are tested to evaluate the physical condition comprehensively. Our studies showed that TG, TC, and LDL in serum followed a gradually increasing trend with increasing fish oil levels, while HDL followed the opposite trend. TG is the major storage form of fatty acid within cells and in circulation [35,36], while TC is the main raw material for the synthesis of various lipid hormones. Similar results were found in *E. fuscoguttatus*♀ × *E. polyphekadion*♂ [29] and Atlantic cod (*Gadus morhua*) [37]. LDL accumulates cholesterol on the walls of blood vessels, which can cause arteriosclerosis, while HDL transports cholesterol to the liver for metabolism and digestion. Previous research showed that a high-lipid diet causes a decrease in HDL and an increase in LDL in *P. sinensis* [18] and blunt snout bream (*M. amblycephala*) [38]. This study also presented similar results. Detection of the above four indicators in the liver of *M. sinensis* was consistent with that in serum. In a healthy state, the liver catabolizes large amounts of fatty acids daily, but only a small amount is stored in the form of TG [39]. This is because the rate of fatty acid acquisition through plasma uptake and resynthesis in the liver is balanced with the rate of fatty acid oxidation and secretion into the plasma in the form of TG. The disruption of metabolic balance leads to increased lipid content in the liver, including triglycerides and cholesterol, which is consistent with a substantial increase in the hepatosomatic ratio in growth performance indicators. GLU is one of the important indexes that reflect the oxidation function of the body using sugars and other nutrients [40,41]. In this study, GLU showed a parabolic pattern with changing oil levels in the diets. *M. sinensis* individuals in group HF-3 reached the highest level in utilizing sugars and lipids compounds to maintain GLU levels. A higher fish oil level did not lead to an increase, which may be due to liver damage. AST, an indicator of liver function, mainly exists in the mitochondria and cytoplasm of liver cells, while ALT is mainly found in the cytoplasm. When the liver is damaged, AST enters the bloodstream [42], along with an increase in the corresponding enzyme activity. The increase in ALT and AST is consistent with the degree of liver cell damage and reflects the health level of the body [43]. In this study, no significant difference in ALT or AST was found in the livers of *M. sinensis* fed with different levels of fish oil (*p* > 0.05); however, ALT and AST in the serum were significantly increased under fish oil supplementation exceeding 11%. Another study also confirmed that elevated ALT and AST levels can cause liver steatosis in mammals [44]. In addition, the results of oil red O staining in the high-level fish oil groups showed swelling of liver cells and significantly increased expression of apoptosis-related genes. These results suggest that excessive lipid levels can cause liver health problems, and the detailed mechanisms need to be explored further.

In this study, the accumulation of lipids in the liver was analyzed through oil red O sections. The positive rate of lipid droplets was positively correlated with the fish oil supplementation level. Most lipids in the body are stored in white adipose tissue, and the liver has the second highest lipid storage capacity, storing lipids mainly in the form of TG [45]. Long-term administration of high-lipid feed can cause lipid droplet formation in the liver, facilitated by dynamic cellular structures that store lipids [46]. When the fish oil supplementation level reaches up to 13.5%, the positive rate of lipid droplets reaches up to 42.76 ± 1.54%. Previous studies reported that high-lipid diets can damage the liver health of pompano (*Trachinotus ovatus*) [47], *E. lanceolatus*♂ *× E. fuscoguttatus*♀ [14], and mice [48]. In *M. sinensis*, a high-lipid diet (fish oil level > 11%) resulted in hepatocyte structural abnormalities (nuclear migration and hepatocyte vacuolation) and liver damage (elevated ALT and AST).

Increasing the supplementation of fish oil in feed increases the pressure of lipid metabolism in the liver [49] and serum [50], which are important components of non-specific immunity. Under normal circumstances, reactive oxygen species generated by oxygen metabolism in organisms are cleared by the antioxidant oxidase system (e.g., SOD, GSH-Px, and CAT) [51]. Under high-lipid feeding conditions, excess lipids in the body are peroxidized, resulting in excessive accumulation of reactive oxygen species [52,53,54] and an increase in MDA levels [55]. In the present study, when the level of fish oil exceeded 8.5%, the MDA levels in the serum and liver were significantly higher than those in the groups supplemented with fish oil levels below 3.5% (i.e., CG and HF-1). More importantly, T-AOC, SOD, GSH-Px, and CAT in the serum and liver decreased in the high-level fish oil groups. According to the oil red O staining results and abnormal physiological and biochemical indexes, the oxidative stress the turtle body experienced induced by a high-lipid diet caused liver damage. Prior research showed that the content of MDA in the liver of *M. salmoides* cultured with a high-lipid diet increased, the antioxidant capacity decreased, and the expression of key genes of cell apoptosis was significantly increased in liver tissue [16]. Similar results were obtained for the blunt snout bream (*M. amblycephala*) [56] and the giant freshwater prawn (*Macrobrachium rosenbergii*) [57] fed with a high-lipid diet. Interestingly, SOD and T-AOC levels in the liver of HF-1 and GSH-Px in HF-2 were significantly increased (*p* < 0.05), both of which were the groups with moderate fish oil levels. This result indicates that a moderate level of lipids (9.08–11.32%) can help stimulate the turtle body to develop a higher antioxidant capacity. Xie et al. pointed out that a certain lipid level provides a protective stimulus to the antioxidant enzyme system of the hybrid grouper [27]. Beyond the limit of adipose-induced oxidative stress, the antioxidant enzyme activity of *M. sinensis* declined sharply, leading to declines in immunity, cell apoptosis, and liver damage; this information can help optimize the lipid level of the feed formula for *M. sinensis*.

Previous studies have shown that the molecular transcription level of antioxidant enzyme genes plays a major regulatory role in resisting oxidative damage induced by dietary lipid levels [58]. Tan et al. found that a high-lipid diet downregulated antioxidant genes (*CAT*, *GSH-Px*, and *GR*) of the hybrid grouper (*E. lanceolatus*♂ *× E. fuscoguttatus*♀) [59]. Zhong et al. found that a high-lipid diet significantly reduced the expression levels of several key genes of antioxidant enzymes (*GPx3*, *GSTO1*, and *CAT*) in *P. sinensis* [18]. In this study, the expression levels of *sod*, *gsh-px*, and *cat* were significantly lower under fish oil levels exceeding 8.5%. These results are consistent with the measured activities of antioxidant enzymes.

The immune status of *M. sinensis* is closely related to the level of its inflammatory factors, which are triggered and regulated by the body’s specific immuno-pro-inflammatory cells and anti-inflammatory factors [60,61]. *IL-6* plays a role in signal transduction in cellular immunity [62], and the concentration of *IL-6* increases under inflammation. *IL-10* and *TGF-β1* play a role in defending against inflammatory responses and balancing pro-inflammatory cytokines [63]. *TNF-α* is produced by macrophages and phagocytes in response to bacterial infection or other immunogenic reactions and promotes cell proliferation and differentiation [64]. *IFN-γ* is the only member of type II interferon, and its functions include the activation of macrophages with antiviral, immunomodulatory, and antitumor properties [14]. *IL-12* is a major cellular pro-inflammatory factor that can act on lymphocytes, which promote the production of *IFN-γ* [65]. In this study, all cytokines except *IL6* were significantly upregulated in the high-level fish oil groups (more than 8.5%). *TNF-α* and *TGF-β* can limit inflammatory responses by regulating other cytokines [66,67], while *IL-12* provides cellular immunity by activating Th1 cells to produce *IFN-γ* [68]. This indicates that *M. sinensis* regulates its cellular immune factors to resist self-injury caused by lipid oxidative stress. The expression level of *IL-6* did not change significantly, which may be related to its function as a signal. Long-term exposure to high lipid stress in *M. sinensis* can play the role of signal transduction, and the transcription level can return to normal.

Apoptosis is the process through which cells automatically end their lives under certain physiological or pathological conditions; apoptosis is controlled by intrinsic genetic mechanisms. The biochemical pathway of cytochrome C release and caspase activation [69,70] is a classic apoptosis pathway. Bax plays a key role in this pathway. When apoptosis is induced, Bax can migrate in membrane receptors such as mitochondria and cell fluid, directly or indirectly forming pores in mitochondria [2]. Cytochrome C is released into the cellular fluid through these pores on the mitochondrial surface; it further binds to apoptosis-related factor 1 to activate caspase-9, which activates other caspases, thereby inducing apoptosis [71]. In contrast, Bcl-2 plays a role in tissue cell apoptosis by releasing cytochrome C [72]. In the present study, when the fish oil level exceeded 8.5%, pro-apoptotic genes were significantly upregulated, and anti-apoptotic genes were significantly downregulated in the liver tissue of *M. sinensis*. This indicates that apoptosis increased in the liver after feeding the turtles with diets high in fish oil. It was also found that in pufferfish (*Takifugu obscurus*) [73] and rats [74], a high-lipid diet can induce apoptosis, similar to the results of the present experiment. Therefore, a reasonable lipid level should be ensured in the diet of *M. sinensis*.

## 5. Conclusions

In this study, with increasing levels of supplemented fish oil, the growth performance of *M. sinensis* first increased and then decreased. The lipid content in the liver and muscle increased. When the level of fish oil supplementation exceeded 8.5%, lipids accumulated in the liver, corroborated by the physiological and biochemical indexes of liver and serum, as well as the results of oil red O staining. Furthermore, liver damage emerged because of the increase in oxidative damage and the induction of liver cell apoptosis, as detected by the activity of antioxidant enzymes and the expression of related genes. These results suggest that a 3.5–6% fish oil level in feed is conducive to improving growth performance under the premise of better immune status.

## Figures and Tables

**Table 1:** Composition and nutrient level of experimental diets (g/kg).

Items	Group					
	CG	HF-1	HF-2	HF-3	HF-4	HF-5
Fish meal	424.0	424.0	424.0	424.0	424.0	424.0
Soybean meal	170.0	170.0	170.0	170.0	170.0	170.0
Wheat flour	200.0	200.0	200.0	200.0	200.0	200.0
Gluten	20.0	20.0	20.0	20.0	20.0	20.0
Fish oil	10.0	35.0	60.0	85.0	110.0	135.0
Choline chloride	2.5	2.5	2.5	2.5	2.5	2.5
Calcium dihydrogen phosphate	15.0	15.0	15.0	15.0	15.0	15.0
Vitamin premix ^a^	1.5	1.5	1.5	1.5	1.5	1.5
Mineral premix ^b^	10.0	10.0	10.0	10.0	10.0	10.0
Sodium carboxymethyl cellulose	10.0	10.0	10.0	10.0	10.0	10.0
Antioxidant	0.5	0.5	0.5	0.5	0.5	0.5
Phagostimulant	0.5	0.5	0.5	0.5	0.5	0.5
Vitamin C ester	1.0	1.0	1.0	1.0	1.0	1.0
Microcrystalline cellulose	135.0	110.0	85.0	60.0	35.0	10.0
Total	1000.0	1000.0	1000.0	1000.0	1000.0	1000.0
Nutrient levels ^c^ (%)						
Crude protein	37.63	37.48	37.54	37.37	37.40	37.34
Crude lipid	6.74	9.08	11.32	13.56	15.99	18.07
Ash	12.53	12.34	12.22	12.04	11.96	11.83
Moisture	8.33	8.25	8.44	8.39	8.56	8.39
Energy (MJ kg^−1^)	17.42	17.77	18.03	18.34	18.67	18.91

^a^ Vitamin premix provided by Hainan Zhengqiang Chaoyue Biochemical Technology Development Co., Ltd. (Haikou, China). The vitamin premix included the following (mg/kg diet): vitamin A (9,000,000 IU), vitamin D_3_ (1,000,000 IU), vitamin E (18,000 IU), vitamin K_3_ (2000 mg), vitamin B_1_ (2000 mg), vitamin B_2_ (5000 mg), vitamin B_6_ (5000 mg), vitamin B_12_ (20 mg), vitamin C (8000 mg), calcium pantothenate (7000 mg), nicotinic acid (18,000 mg), folic acid (300 mg), and biotin (45 g). ^b^ The mineral premix included the following (mg/kg diet): CuSO_4_·5H_2_O (2 g), ZnSO_4_·H_2_O (25 g), MnSO_4_·H_2_O (15 g), FeSO_4_·H_2_O (25 g), KI (0.04 g), CoSO_4_·7H_2_O (0.1 g), and Na_2_SeO_3_ (0.02 g). ^c^ The nutrient level was measured.

**Table 2:** Primer information of real-time fluorescence quantitative PCR.

Target Gene	Forward Primer (5′–3′)	Reverse Primer (5′–3′)	Genbank Number	Primer Efficiency (%)
*Bcl-2*	ATCCAAGACAACGGAGGCTG	CAGATAAGCGCCAAGGGTGA	XM_034762099.1	98.04
*Caspase-3*	GGACTGCAATCAGGTCAAGA	CTGGCTGTATTCCAGAGTCC	XM_024194340.1	96.65
*BAX*	ATCAACGTCTTCGTGGGGTC	ATGATGAGCTGGGTGTCGAA	XM_034792450.1	93.46
*mn-sod*	GGGTCACATCAACCACACCA	AAAGGAGCCAAAGTCACGCT	XM_034765292.1	98.04
*cat*	GGCATTGAACCTAGCCCTGA	GTCCTAAACGGTGTCGGTGA	XM_034769697.1	94.46
*gsh-px*	TCCCTATAGACCGGTCCTGC	TCCCCTGAGATTTCCCCTGT	XM_005295795.5	93.07
*IFN-γ*	CCCGGCTACAAGTAAATCGC	GCTTTCAGTTAGGCTGTCGTTC	XM_034754901.1	92.44
*TNF-α*	TCCATTCCTCTCCGGCATAC	AGATGGACTCGAACCACACC	XM_008176809.1	99.95
*TGF-β1*	GTCGCTCTACAACAGCACCC	TGCACCTCCTTGGCGT	XM_005310533.2	93.30
*IL-6*	CCTCCCCAAGATCACAGAGG	CTGGAGATTCCGCTCAAGCA	XM_008170880.2	94.86
*IL-10*	TGCTGGACCTGAAGCAGACA	ATGGTTTGGTTCCTCTTCTCGC	XM_034769759.1	92.28
*IL-12*	AGCAAGTCAGAAGCAAGAGG	ATAATCTGCCTTGGGGAGGAC	XM_005300109.2	99.08
*β-actin*	GCACCCTGTGCTGCTTACA	CACAGTGTGGGTGACACCAT	XM_039490283.1	98.16

Note: *Bcl-2*: B-cell lymphoma-2; *Caspase-3*: cysteinyl aspartate-specific proteinase-3; *BAX*: Bcl2-Associated X; *Mn-SOD*: extracellular manganese superoxide dismutase; *CAT*: catalase; *GSH-Px*: glutathione peroxidase; *IFN-γ*: interferon-γ; *TNF-α*: tumor necrosis factor-α; *TGF-β1*: transforming growth factor-β1; *IL-6*: interleukin 6; *IL-10*: interleukin 10; *IL-12*: interleukin 12.

**Table 3:** Effect of fish oil level on the growth performance of *M. sinensis*.

	CG (1%)	HF-1 (3.5%)	HF-2 (6%)	HF-3 (8.5%)	HF-4 (11%)	HF-5 (13.5%)
IW/g	65.32 ± 0.53	65.44 ± 0.35	64.78 ± 0.30	65.29 ± 0.33	65.60 ± 0.32	65.50 ± 0.43
FW/g	102.99 ± 0.98 ^a^	113.59 ± 0.76 ^b^	119.08 ± 0.50 ^c^	127.45 ± 0.52 ^d^	119.40 ± 0.35 ^c^	118.28 ± 0.99 ^c^
SR %	100	100	100	100	100	100
WGR %	57.69 ± 2.21 ^a^	73.59 ± 0.41 ^b^	83.83 ± 1.23 ^c^	95.22 ± 0.73 ^d^	82.03 ± 0.54 ^c^	80.59 ± 1.25 ^c^
SGR %	0.65 ± 0.020 ^a^	0.79 ± 0.003 ^b^	0.87 ± 0.010 ^c^	0.96 ± 0.005 ^d^	0.86 ± 0.004 ^c^	0.84 ± 0.010 ^c^
FCR	1.82 ± 0.026 ^e^	1.62 ± 0.015 ^d^	1.51 ± 0.017 ^b^	1.45 ± 0.009 ^a^	1.56 ± 0.006 ^bc^	1.59 ± 0.024 ^cd^
HSI %	8.37 ± 0.38 ^a^	9.05 ± 0.34 ^ab^	9.16 ± 0.41 ^ab^	9.76 ± 0.21 ^bc^	10.44 ± 0.17 ^c^	9.78 ± 0.16 ^bc^
VSI %	19.26 ± 0.69 ^a^	20.36 ± 0.49 ^a^	22.83 ± 0.41 ^b^	22.94 ± 0.16 ^b^	23.83 ± 1.43 ^b^	23.16 ± 0.17 ^b^

Note: Different letters at the corner of the same row indicate significant differences (*p* < 0.05).

**Table 4:** Effects of fish oil level on the muscle composition of *M. sinensis* (*n* = 9).

Group	Moisture, %	Ash, %	Crude Protein, %	Crude Lipid, %
CG (1%)	80.79 ± 0.51	6.87 ± 0.37 ^ab^	81.75 ± 0.60 ^c^	8.23 ± 0.50 ^a^
HF-1 (3.5%)	80.49 ± 0.23	7.2 ± 0.13 ^b^	81.36 ± 1.20 ^bc^	9.12 ± 0.32 ^ab^
HF-2 (6%)	79.9 ± 0.45	6.82 ± 0.40 ^ab^	80.81 ± 0.37 ^abc^	9.73 ± 0.34 ^bc^
HF-3 (8.5%)	80.75 ± 0.15	6.69 ± 0.23 ^ab^	79.51 ± 0.19 ^ab^	10.51 ± 0.45 ^c^
HF-4 (11%)	80.52 ± 0.22	6.59 ± 0.20 ^ab^	79.09 ± 0.44 ^a^	11.94 ± 0.16 ^d^
HF-5 (13.5%)	80.86 ± 0.34	6.33 ± 0.16 ^a^	79.31 ± 0.35 ^a^	11.81 ± 0.18 ^d^

Note: Different letters at the corner of the same column indicate significant differences (*p* < 0.05).

**Table 5:** Effect of fish oil level on the serum physiological and biochemical indexes of *M. sinensis* (*n* = 9).

	CG (1%)	HF-1 (3.5%)	HF-2 (6%)	HF-3 (8.5%)	HF-4 (11%)	HF-5 (13.5%)
Blood lipid	-	-	-	-	-	-
TG (mmol/L)	1.88 ± 0.09 ^a^	4.94 ± 0.52 ^bc^	4.15 ± 0.03 ^b^	5.79 ± 0.02 ^cd^	6.30 ± 0.50 ^d^	6.39 ± 0.32 ^d^
TC (mmol/L)	3.74 ± 0.10 ^a^	3.82 ± 0.03 ^a^	4.14 ± 0.03 ^a^	4.81 ± 0.38 ^b^	5.16 ± 0.07 ^b^	5.28 ± 0.17 ^b^
HDL (mmol/L)	1.52 ± 0.02 ^c^	1.46 ± 0.14 ^c^	1.38 ± 0.11 ^c^	1.14 ± 0.03 ^b^	0.86 ± 0.01 ^a^	1.01 ± 0.03 ^ab^
LDL (mmol/L)	1.70 ± 0.04 ^ab^	1.55 ± 0.02 ^a^	1.74 ± 0.02 ^b^	1.99 ± 0.12 ^c^	2.21 ± 0.04 ^d^	2.24 ± 0.02 ^d^
Blood glucose	-	-	-	-	-	-
GLU (mmol/L)	3.57 ± 0.09 ^a^	3.70 ± 0.15 ^ab^	4.07 ± 0.15 ^cd^	4.33 ± 0.03 ^d^	4.00 ± 0.06 ^bc^	3.73 ± 0.03 ^ab^
Liver function	-	-	-	-	-	-
ALT (U/L)	4.03 ± 0.04 ^a^	5.11 ± 2.01 ^a^	5.67 ± 0.33 ^ab^	6.14 ± 0.3 ^ab^	8.25 ± 0.58 ^bc^	10.33 ± 0.33 ^c^
AST (U/L)	65.00 ± 0.58 ^ab^	58.33 ± 0.67 ^a^	77.00 ± 8.08 ^b^	94.33 ± 0.88 ^c^	92.33 ± 3.28 ^c^	94.67 ± 4.33 ^c^
TP (g/L)	21.93 ± 0.24 ^c^	20.6 ± 0.23 ^b^	22.47 ± 0.24 ^c^	29.77 ± 0.63 ^d^	19.90 ± 0.10 ^b^	15.97 ± 0.03 ^a^
ALB (g/L)	8.37 ± 0.07 ^e^	7.53 ± 0.09 ^c^	8.07 ± 0.13 ^d^	10.83 ± 0.07 ^f^	7.10 ± 0.01 ^b^	5.67 ± 0.03 ^a^
GLO (μmol/L)	13.57 ± 0.18 ^bc^	13.07 ± 0.15 ^b^	14.40 ± 0.12 ^c^	18.93 ± 0.62 ^d^	12.80 ± 0.10 ^b^	10.30 ± 0.06 ^a^
TBIL (μmol/L)	0.80 ± 0.30	0.67 ± 0.22	0.83 ± 0.22	1.17 ± 0.07	0.77 ± 0.27	0.80 ± 0.31
DBIL (μmol/L)	0.10 ± 0.01 ^a^	0.20 ± 0.06 ^ab^	0.23 ± 0.07 ^ab^	0.27 ± 0.07 ^b^	0.30 ± 0.06 ^b^	0.33 ± 0.07 ^b^
I-BIL (/L)	0.70 ± 0.30	0.47 ± 0.27	0.57 ± 0.19	0.87 ± 0.09	0.43 ± 0.23	0.57 ± 0.24
r-GT (U/L)	1.67 ± 0.88 ^a^	2.00 ± 0.58 ^a^	4.67 ± 1.67 ^ab^	7.33 ± 0.88 ^bc^	11.33 ± 2.33 ^c^	11.33 ± 2.40 ^c^

Note: Different letters at the corner of the same row indicate significant differences (*p* < 0.05).

**Table 6:** Effect of fish oil level on the liver physiological and biochemical indexes of *M. sinensis* (*n* = 9).

	CG (1%)	HF-1 (3.5%)	HF-2 (6%)	HF-3 (8.5%)	HF-4 (11%)	HF-5 (13.5%)
TG (mmol/gprot)	1.53 ± 0.10 ^a^	1.72 ± 0.06 ^ab^	1.81 ± 0.11 ^b^	1.82 ± 0.04 ^b^	1.80 ± 010 ^b^	1.81 ± 0.06 ^b^
TC (mmol/gprot)	0.94 ± 0.03 ^a^	0.82 ± 0.04 ^a^	1.10 ± 0.05 ^b^	1.10 ± 0.03 ^b^	1.18 ± 0.04 ^b^	1.17 ± +0.10 ^b^
HDL (mmol/gprot)	0.45 ± 0.01 ^c^	0.45 ± 0.01 ^c^	0.41 ± 0.01 ^b^	0.41 ± 0.01 ^b^	0.38 ± 0.01 ^a^	0.41 ± 0.01 ^b^
LDL (mmol/gprot)	0.41 ± 0.02 ^a^	0.43 ± 0.01 ^ab^	0.47 ± 0.02 ^ab^	0.47 ± 0.03 ^ab^	0.50 ± 0.03 ^b^	0.58 ± 0.04 ^c^
ALT (U/gprot)	10.58 ± 3.05	5.10 ± 3.51	6.76 ± 1.33	12.84 ± 3.22	13.52 ± 0.84	13.92 ± 3.43
AST (U/gprot)	19.42 ± 9.34	19.00 ± 1.44	30.53 ± 1.01	26.95 ± 4.15	22.68 ± 4.93	34.48 ± 3.04

Note: Different letters at the corner of the same row indicate significant differences (*p* < 0.05).

## Data Availability

The partial data analyzed in this study are available from the corresponding author upon reasonable request.
